# Protein-protein conjugate nanoparticles for malaria antigen delivery and enhanced immunogenicity

**DOI:** 10.1371/journal.pone.0190312

**Published:** 2017-12-27

**Authors:** Puthupparampil V. Scaria, Beth Chen, Christopher G. Rowe, David S. Jones, Emma Barnafo, Elizabeth R. Fischer, Charles Anderson, Nicholas J. MacDonald, Lynn Lambert, Kelly M. Rausch, David L. Narum, Patrick E. Duffy

**Affiliations:** 1 Laboratory of Malaria Immunology and Vaccinology, NIAID, National Institutes of Health, Rockville, Maryland, United States of America; 2 EM Unit/RTB Rocky Mountain Laboratories/NIAID/NIH, Hamilton, MT, United States of America; Ehime Daigaku, JAPAN

## Abstract

Chemical conjugation of polysaccharide to carrier proteins has been a successful strategy to generate potent vaccines against bacterial pathogens. We developed a similar approach for poorly immunogenic malaria protein antigens. Our lead candidates in clinical trials are the malaria transmission blocking vaccine antigens, Pfs25 and Pfs230D1, individually conjugated to the carrier protein Exoprotein A (EPA) through thioether chemistry. These conjugates form nanoparticles that show enhanced immunogenicity compared to unconjugated antigens. In this study, we examined the broad applicability of this technology as a vaccine development platform, by comparing the immunogenicity of conjugates prepared by four different chemistries using different malaria antigens (PfCSP, Pfs25 and Pfs230D1), and carriers such as EPA, TT and CRM197. Several conjugates were synthesized using thioether, amide, ADH and glutaraldehyde chemistries, characterized for average molecular weight and molecular weight distribution, and evaluated in mice for humoral immunogenicity. Conjugates made with the different chemistries, or with different carriers, showed no significant difference in immunogenicity towards the conjugated antigens. Since particle size can influence immunogenicity, we tested conjugates with different average size in the range of 16–73 nm diameter, and observed greater immunogenicity of smaller particles, with significant differences between 16 and 73 nm particles. These results demonstrate the multiple options with respect to carriers and chemistries that are available for protein-protein conjugate vaccine development.

## Introduction

The complex life cycle of the *Plasmodium* parasite presents multiple targets to design therapeutics and vaccines against individual stages or multiple stages simultaneously, to interrupt the life cycle [1]. While most efforts have focused on pre-erythrocytic and blood stage vaccines against *Plasmodium falciparum* [2–5], there has been increasing interest in sexual stage vaccines, also termed transmission-blocking vaccines (TBVs), which could play a role in malaria elimination strategies [6–9]. Humans vaccinated with TBV antigens generate antibodies which are ingested by the mosquito during a blood meal and can block development of the parasite in the midgut and prevent further transmission. A few antigens, expressed in gametocytes or zygotes, have been identified as candidates for TBV [6, 9–17]. Among the TBV antigens under vaccine development, Pfs230 and Pfs48/45 are expressed in gametocytes, whereas Pfs25 is expressed on zygotes and ookinetes within mosquitoes. These proteins are stabilized by multiple disulfide bonds; hence their manufacturing has been challenging [18–20], and the resulting antigens have been relatively small and poorly immunogenic.

One strategy to enhance vaccine responses is to conjugate a poorly immunogenic molecule to a strongly immunogenic protein or particulate carrier. This approach has been extremely successful in developing polysaccharide conjugate vaccines, where the T-independent immunogenicity of poorly immunogenic polysaccharide antigens is transformed to T-dependent immunogenicity, generating boostable vaccines against a variety of bacterial infections [21]. Current licensed polysaccharide conjugate vaccines use Tetanus Toxoid (TT) [22], Diphtheria Toxoid [23], CRM197 [24], Protein D [25] or Outer Membrane Protein Complex (OMPC) [26] as carriers. These platforms, in addition to providing T cell epitopes for T cell recruitment, provide a particulate structure that allows efficient internalization of the immunogen by antigen-presenting cells (APC) and in some cases, allows multivalent presentation of antigens on the surface that facilitates B cell receptor cross-linking and stimulation. Conjugation has also been employed to make poorly immunogenic protein subunit and peptide antigens more immunogenic by conjugation to antigen delivery platforms like virus-like particles [27–33], nanoparticles [34–36], membrane vesicles [37, 38] and protein conjugates [39–41] for malaria vaccine development. Though no protein-protein conjugate vaccine has been approved for human use, the most advanced malaria vaccine, RTS,S, is a particulate vaccine with the antigen (partial sequence of *P*. *falciparum* circumsporozoite protein (CSP)) fused to a carrier protein (hepatitis B virus surface antigen) that forms a self-assembled VLP [42–44].

Our laboratory has pioneered conjugation of malaria antigens to protein carriers to generate effective immunogens and potential vaccines [45–49]. Chemical conjugation of malaria antigens to carrier proteins generates nanoparticles with enhanced immunogenicity [45,49], and some of these have progressed to clinical evaluation [50]. The lead antigens in our clinical trials are Pfs25 and Pfs230, TBV antigens expressed in zygotes/ookinetes and gametocytes, respectively. Chemical conjugation of these antigens to the recombinant Exoprotein A (EPA) carrier increased functional immunogenicity in animal studies, and the conjugates have proven to be safe in human trials [50]. While these studies focused on EPA conjugates prepared by thioether chemistry [45,49], we are also exploring this platform technology with a variety of malaria antigens and carriers. For example, we find that both carriers and adjuvants can influence the level of immune response against conjugated malaria antigens [48]. Here we have examined the immunogenicity of conjugated antigens using a number of antigen-carrier conjugates prepared by different synthetic chemistries, and demonstrated the broad utility of this technology for vaccine development.

## Materials and methods

### Antigens and carriers

The carrier protein recombinant Exoprotein A (rEPA or EPA) of *Pseudomonas aeruginosa* (molecular weight, 66,983 Da) was expressed in *E*. *coli* [47]. Recombinant antigens Pfs25 (mol. Wt. 18,735) [51], Pfs230D1 (amino acids Ser^542^- Gly^736^ of domain-1 of Pfs230 with mol. wt. 21,854) [14] and PfCSP (*P*. *falciparum* CSP from Gly^86^ to Ser ^411^, with mol. wt. 33,079) [52] were based on *P*. *falciparum* 3D7 allele sequence and were codon optimized and produced in *P*. *Pastoris*, as described previously. TT and CRM197 were obtained from GSK Vaccines S.r.l. (Siena, Italy).

### Chemicals and reagents

*N*-(3-Dimethylaminopropyl)-*N*′-ethylcarbodiimide hydrochloride (EDC), N-Hydroxysulfosuccinimide (sNHS), Adipic acid dihydrazide (ADH) and a 25% solution of Glutaraldehyde (GA) in water were obtained from Sigma-Aldrich (St. Louis, MO). N-(ε-maleimidocaproyloxy)succinimide (EMCS), N-(ε-maleimidocaproyloxy)sulfo-succinimide sodium salt (sulfo-EMCS), and S-acetylthioglycolic acid N-hydroxysuccinimidyl ester (SATA) were purchased from Pierce Biotechnology Inc. (Rockford, IL). CFD10 and CFD100 Amicon centrifugal filtration devices with 10 kDa and 100 kDa MWCO respectively were obtained from Millipore (Billerica, MA).

### Syntheses of conjugates

#### Thioether Chemistry (TE)

Buffer A: pH 7.2 PBSE (100 mM sodium phosphate, 150 mM NaCl, 5 mM EDTA); Buffer B: pH 6.5 PBSE (100 mM sodium phosphate, 150 mM NaCl, 5 mM EDTA); Buffer C: pH 7.4 PBS for chromatography (1X PBS, pH 7.4); Deacetylation Buffer = 0.5 M NH_2_OH in pH 7.2 PBSE.

Conjugation of various antigens to carrier proteins by thioether chemistry was carried out by a procedure similar to the one described below for Pfs230D1-EPA as an example. All reactions, including the modification of antigen and carrier, as well as conjugation of the modified proteins were carried out in a constant temperature bath at 22°C.

#### Maleimide-activated rEPA (rEPA-Mal_11_)

A solution containing 19.23 mg of rEPA in 6.2 mL of pH 7.2 PBSE (3.1 mg/mL, 2.87 x 10^−7^ mol.) was equilibrated at 22°C. To this solution, 287 μL of a 30.8 mg/mL solution of EMCS in DMSO (8.85 mg, 2.87 x 10^−5^ mol) was added and the mixture was stirred for 90 min at 22°C. The reaction mixture was immediately exchanged into pH 6.5 PBSE using a CFD10 to obtain 4.5 mL of maleimide-activated rEPA. An indirect DTDP assay determined the number of maleimides per molecule of rEPA to be 11. The protein concentration was determined to be 4.23 mg/mL using an extinction coefficient (E_280_ = 87,001 L mol^-1^ + 515 L mol^-1^/mole of maleimide groups attached) adjusted for maleimide contribution. The yield of rEPA-(mal)_11_ was 19 mg (98%).

#### Sulfhydryl-modified Pfs230D1 (Pfs230D1-(SH)_3.8_)

A solution containing 9.5 mg (4.35 x 10^−7^ mol) of Pfs230D1 in 4.75 mL of pH7.2 PBSE was stirred at 22°C, and 43.7 μL (1.0 mg, 4.35 X 10^−6^ mol) of a 23 mg/mL solution of SATA in DMSO was added to the solution. The resulting solution was stirred for 1 hour at 22°C. The mixture was diluted with pH7.2 PBSE and concentrated repeatedly to have a 1000-fold buffer exchange using a CFD10 to a final volume of approximately 4 mL (approximately 2 mg/mL). The resulting solution (4 ml) was combined with 0.4 mL of deacetylation buffer (0.5M NH_2_OH in pH7.2 PBSE) and placed on a rotating shaker at 600 RPM for 1 hour at room temperature. The mixture was transferred to a CFD10, and the buffer was exchanged with pH6.5 PBSE buffer by repeated dilution and concentration to have a 1000-fold buffer exchange, yielding 3.5 mL of a 2.57 mg/mL solution of SH-modified Pfs230M (8.996 mg). The product was kept frozen at -80°C. Determination of thiol by DTDP assay gave 3.8 thiols per molecule of Pfs230D1. The yield of Pfs230D1-(SH)_3.8_ was 8.996 mg (98.8%).

#### Pfs230D1-rEPA conjugate

3.4 ml of 2.57 mg/ml Pfs230D1-SH_3.8_ was concentrated to 0.85 ml of 9.66 mg/ml and 2.25 ml of 4.23 mg/ml rEPA-mal_11_ was concentrated to 1.1 ml of 7.84 mg/ml using a CFD10.

To 0.8 mL of a 7.84 mg/mL solution of rEPA-(mal)_11_ in pH 6.5 PBSE (6.27 mg, 9.36 x 10^−8^ mol), with stirring in a 22°C water bath, 1.2 ml of same buffer was added first, followed by 0.8 mL of a 9.66 mg/mL solution of Pfs230D1(SH)_3.80_ (7.73 mg, 3.54 x 10^−7^ mol) in pH 6.5 PBSE. The mixture was stirred for 1 hour at 22°C. Excess maleimide groups were quenched by adding 9.2 mL of a 20 mg/mL solution of cysteine hydrochloride (0.184 mg, 1.04 x 10^−6^ mol) in pH 6.5 PBSE, and the mixture was stirred for an additional 15 minutes. Purification was accomplished by fractionation on a 16 mm x 60 cm Superdex 200 column at 1 mL/min in PBS solution. One mL fractions were collected across the protein peak as detected at 280 nm. Fractions eluting from 50–73 minutes were combined to give 7.14 mg of product at a concentration of 2.07 mg/mL, as determined by absorbance at 280 nm. Final product was analyzed by size exclusion chromatography (G5000PWxl column) and gel electrophoresis (3–8% Tris acetate SDS-PAGE). Average molecular mass based on SEC MALS was 648 kDa and antigen/carrier molar ratio, based on amino acid analysis, was 2.53 (45.2% Pfs230M by mass). Extinction coefficient, E^0.1%^_280,_ of this conjugate, calculated from amino acid analysis was 1.056 mg^-1^ ml.cm^-1^ and the overall yield based on Pfs230D1 was 42%.

#### Amide Method (Am)

The following buffers were used for the conjugation reaction based on amide method of conjugation:

Buffer A = pH 6.5 MES: pH6.5 (50 mM 2-(*N*-morpholino) ethanesulfonic acid (“MES”), 150 mM NaCl)

Buffer B = 100 mM PBS pH8.0; Buffer C = 4 mM PBS pH7.4

#### Synthesis of Pfs230D1-EPA conjugate by amide chemistry

5.00 mg (2.29X10^-7^ moles) of Pfs230D1 in 0.62 ml solution of Buffer A was mixed with 4.09 mg (6.11X10^-8^ moles) of EPA in 0.34 ml of Buffer A. To this protein mixture was added 0.017 ml of Buffer A and 9.22 μl of a 230 mM Sulfo-NHS solution (0.461 mg, 2.12X10^-6^ moles) in Buffer A. Following a quick vortex, 21.2 μl of a 1M EDC solution (4.07 mg, 2.12X10^-5^ moles) in Buffer A was added to the reaction mixture. The final protein concentration was 9.00 mg/ml in 2.1 mM Sulfo-NHS and 21 mM EDC. The resulting reaction was mixed for 3¼ hours in an orbital shaker at 800 RPM at ~25°C. Following this reaction, 1.5 ml of Buffer B was added to the entire reaction and allowed to continue mixing at 800 RPM at for 90 min. The quenched reaction was then concentrated and was purified by fractionation using a HiLoad™ 16/60 Superdex™ 200 preparative column at 1 ml/min in Buffer C. One ml fractions were collected across the protein peak as detected by absorbance at 280 nm. Fractions eluting from 49–73 min were pooled and concentrated using a CFD100 to a volume of 2.5 ml in Buffer C. The retentate was syringe-filtered through a 0.22 μm PVDF membrane. Final product was analyzed by SEC-HPLC on a G5000PW_XL_ column with MALS/dRI detectors. Weighted mass average (Mw) pf the conjugate was determined to be 690 kDa. Amino acid analysis determined the molar ratio of Pfs230D1:EPA to be 2.08:1, with the fractional mass of Pfs230D1 equal to 0.405. The extinction coefficient, E^0.1%^_280,_ of this conjugate, calculated from amino acid analysis was 1.081 mg^-1^·ml·cm^-1^. The total protein concentration was determined by A_280_ to be 2.036 mg/ml, with the protein concentration of Pfs230D1 equal to 0.824 mg/ml. Recovery of Pfs230D1 for this prep was calculated to be 2.07 mg, with an overall yield of 41%.

#### Adipic Acid Dihydrazide (ADH)-mediated coupling

The following buffers were used for the ADH-mediated conjugation of PfCSP to rEPA:

Buffer A = pH4.7 MES (100 mM 2-(*N*-morpholino) ethane sulfonic acid (“MES”), 150 mM NaCl)

Buffer B = 0.2M ADH in buffer A, adjusted to pH4.7

Buffer C = 4 mM PBS pH 7.4.

#### ADH modification of rEPA

To a 9.2 ml solution of 9.5 mg (1.42 X10^-7^ moles) of rEPA in Buffer B containing 0.2M ADH (320.53 mg, 1.84 X10^-3^ moles), 37 μl of 1M EDC (7.1 mg, 3.70 X10^-5^ moles) was added and mixed. The final protein concentration was 1.034 mg/ml in 0.2M ADH and 4mM EDC. The resulting reaction was stirred in a 50 ml conical tube in a 22^°^C water bath at 600 RPM for 2 hours. The mixture was then dialyzed into Buffer A using a 15 ml CFD10 centrifugal dialysis device with 10K molecular weight cut off. 9.18 mg rEPA-AH were obtained, a protein recovery of 96.5%. Based on TNBS assay, the number of hydrazides per rEPA was 17.0.

#### Conjugation of PfCSP with rEPA-AH

To a solution of 4.59 mg (6.85 x 10^−8^ moles) of rEPA-AH_**17**_ in 1.15 ml Buffer A, the following was added: 3.928 mg (1.207 x 10^−7^ moles) of PfCSP in 0.545 ml of Buffer A, and 8.6 ul of 1M EDC; the mixture was stirred at 22^°^C in a water bath at 300 RPM for 120 min. The resulting solution was desalted by a PD10 column and was purified by fractionation on a 16/60 Superdex 200 preparative grade column at 1.0 ml/min in Buffer C. One ml fractions were collected across the protein peak as detected by absorbance at 280 nm. Protein containing fractions were pooled and concentrated using CFD10 to a final volume of 3 ml in Buffer C. The 3 ml pool was syringe-filtered through a 0.22μm PVDF membrane. The final product was 2.8 ml. It was analyzed by SEC-HPLC on a G5000PW_XL_ column with a MALS/dRI detector, and the weighted mass average (Mw) was determined to be 654 kDa. The AAA results showed the protein concentration = 1.11 mg/ml. The extinction coefficient, E^0.1%^_280,_ of this conjugate, calculated from amino acid analysis was 0.873 mg^-1^·ml·cm^-1^. The yield based on PfCSP for this preparation was calculated to be 1.29 mg (33%).

#### Glutaraldehyde crosslinking (GA)

Following buffers were used for the glutaraldehyde mediated crosslinking reaction. Buffer A = PBS pH7.0 (100 mM Sodium Phosphate, 150 mM NaCl); Buffer B = 1 M L-Lysine in Buffer A (prepared with Sigma #L-9037); Buffer C = 4 mM PBS pH7.4 (prepared with 10X PBS, Gibco #70011)

#### Conjugation of PfCSP with rEPA

To a 120 μl solution of 0.884 mg (2.71 X10^-8^ moles) of PfCSP and 0.736 mg (1.10 X10^-8^ moles) of EPA in Buffer A was added 38.4 μl of Buffer A and 3.56 μl of a 25% solution of glutaraldehyde in water (Sigma G5882, 0.890 mg, 8.89 X10^-6^ moles). The final protein concentration was 10.0 mg/ml in 0.55% (w/v) Glutaraldehyde. The resulting reaction was mixed for 150 minutes in an orbital shaker at 1,200 RPM at 22°C. The reaction was then quenched by the addition of 17.8 μl of Buffer B and the continued mixing as before for 60 minutes. The quenched reaction was then diluted to 1.00 ml with Buffer A and purified by fractionation on a HiLoad™ 16/60 Superdex™ 200 prep grade column at 1 ml/min in Buffer C. One ml fractions were collected across the protein peak as detected by absorbance at 280 nm. Fractions eluting from 45–66 min post-injection were pooled and concentrated using a CFD100 to a volume of 0.84 ml in Buffer C. The retentate was syringe-filtered through a 0.22μm PVDF membrane to a final volume of 820 μl. The final product was analyzed by SEC-HPLC on a G5000PW_XL_ column with MALS/dRI detector. The weighted mass average (Mw) was determined to be 797 kDa. The AAA results showed the molar ratio of PfCSP/EPA to be 1.60 with the fractional mass of PfCSP equal to 0.438. The extinction coefficient, E^0.1%^_280,_ of this conjugate, calculated from amino acid analysis was 0.848 mg^-1^·ml·cm^-1^. The yield based on PfCSP for this prep was 0.338 mg (38%).

#### Determination of thiol and maleimide modifications

Concentration of thiol moieties in the thiol modified proteins were determined by 4,4’-dithiodipyridine (DTDP) assay. Thiolated proteins were incubated with DTDP at pH 6.5 which, on reaction with sulfhydryl groups, releases a molar equivalent of 4-thiopyridon. Concentration of 4-thiopyridon was determined from the absorbance at 324 nm [53], using a molar extinction coefficient of 21,400 L M^-1^. The assay result was validated by cysteine standards routinely included in the assay.

The number of maleimide moieties in the maleimide modified proteins was determined by a “reverse-DTDP” assay. In this assay, the DTDP assay is used to determine the amount of cysteine consumed by maleimide moieties, when incubated with known amounts of cysteine. Maleimide-modified proteins were mixed with standard cysteine solutions, and the mixtures were incubated for 1 hour at room temperature. Thiol concentrations were measured as described above using the DTDP assay. Maleimide concentration was calculated as the difference between the thiol concentration of the cysteine solution and the cysteine plus maleimide-modified protein.

#### Conjugate analysis

Conjugates were analyzed by size exclusion chromatography using a G5000PWxl (Tosoh Biosciences, King of Prussia, PA) column, on an Agilent 1100 HPLC coupled to multi-angle light scattering (SEC-MALS) and Quasi-elastic light scattering (QELS) (Wyatt DAWN HELIOS II and QELS, Wyatt Technologies, Santa Barbara, CA) detectors. The average molar mass (Mw), molecular weight distribution and hydrodynamic radius (Rh) of the conjugates were determined using ASTRA software.

#### Determination of protein composition

Composition of the conjugates was determined from amino acid analyses of the conjugates and individual protein components (antigen and carrier) following the method as previously described [54]. The molar ratios of the proteins in the conjugates were calculated from the amino acid analysis data, by a multiple regression analysis with least square fitting, of a selected set of amino acids common to both proteins [54]. Amino acid analyses were performed at the W.M. Keck Biotechnology Resource Lab at the Yale School of Medicine (New Haven, CT).

#### Immunogenicity studies

Immunogenicity of various conjugates were evaluated in CD-1 mice (Charles River Laboratories). Groups of 10 mice were vaccinated on Days 0 and 28 by intra-muscular injection of 50 μl formulations containing the stated dose for each animal. Blood samples from animals were collected either from submandibular vein for intermediate time points (25 μl) or by cardiac puncture, after anesthesia, for final bleed (1 ml). Samples were spun down to obtain sera and were analyzed for antigen-specific antibody titer by ELISA. All animal studies were carried out per the guidelines and approval of Animal Care and Use committee at the National Institutes of Health.

#### Antibody levels and analysis

Antigen-specific antibody titers were assayed using standardized ELISA with respective antigens as the plate antigens [55]. ELISA units were determined from the absorbance relative to reference antisera against the respective antigens generated in mice immunized with the un-modified antigen.

#### Statistical analysis

ELISA data were analyzed with Prism software (GraphPad Software, Inc., La Jolla, CA) and statistical differences between groups (P ≤ 0.05) were measured using a Kruskal-Wallis analysis followed by a Dunn multiple comparator test for comparing three or more groups.

#### Scanning electron microscopy

25 microliters of solution were aliquoted on silicon chips and allowed to settle for 15 minutes. The particles were then fixed with 2.5% glutaraldehyde in 0.1 M sodium cacodylate buffer, and post-fixed with 1.0% osmium tetroxide in 0.1 M sodium cacodylate buffer. Specimens were dehydrated with a graded ethanol series, critical point dried under CO_2_ in a Bal-Tec model cpd 030 Drier (Balzers, Liechtenstein), mounted on aluminum studs, and sputter coated with 50 A of iridium in a model IBSe ion beam sputter coater (South Bay Technologies, San Clemente, CA) prior to viewing at 5 kV in a Hitachi SU-8000 field emission scanning electron microscope (Hitachi, Tokyo, Japan).

#### Transmission electron microscopy

5 microliter droplets were aliquoted on freshly glow discharged 200 mesh carbon coated copper grids and allowed to settle for one minute. Excess was wicked away with filter paper points and 5 microliters of NanoVan stain was added for 2 minutes and then excess was wicked away (Nanoprobes, Inc, Yaphank, NY). After drying, samples were viewed at 80 kV on a Hitachi 7500 transmission electron microscope (Hitachi, Tokyo, Japan). Digital images were acquired with an AMT digital camera system (AMT, Chazy, NY).

## Results and discussion

A variety of conjugation chemistry options are available for conjugating a protein or peptide to another protein. These include small homo- or hetero-bifunctional cross-linkers with varying spacer lengths that can create linkages between amine, carboxyl and sulfhydryl groups. To generate protein-protein conjugates of antigen to carrier, we examined: 1) EDC-mediated coupling between carboxylic acid from one protein and amine from the second protein, 2) ADH chemistry where a carboxyl from one protein is modified with ADH and allowed to couple to a carboxyl from a second protein by EDC coupling, 3) thioether chemistry where a thioether bond is generated by coupling a sulfhydryl-modified amino group of one protein to a maleimide-modified amino group from the second protein and 4) glutaraldehyde mediated cross linking. Since multiple functional groups are randomly modified by the cross-linking agents, often used in excess, conjugates will have more than one covalent bond linking them, generating macromolecular cross-linked structures with nanoparticle dimensions.

### Effect of conjugation chemistry

Conjugation chemistry can influence the characteristics of macromolecular structures formed and thus can affect the immunogenicity of conjugates. We compared conjugates prepared using four different chemistries to assess any differences in immunogenicity due to conjugation chemistry. For initial evaluation of chemistry, we synthesized EPA conjugates of PfCSP (a pre-erythrocytic antigen) by four different chemistries. Conjugates on SDS-PAGE appear as high molecular weight smeared bands (S1A Fig) reactive to an anti-PfCSP monoclonal antibody, 1G2, on a Western blot (S1B Fig), confirming the presence of PfCSP. SEC-MALS analysis was used to determine the average molecular weight, molecular weight distribution and particle size, as described by Jones et al [45]. Table 1 shows the physico-chemical parameters of various conjugates of PfCSP, Pfs25 and Pfs230D1 described in this report.

**Table 1:** Physico-chemical parameters of various conjugates of PfCSP, Pfs25 and Pfs230D1 with EPA, TT or CRM197.

Conjugate	Lot #	Conjugation Chemistry	Mol. Wt. distribution (kDa)^a^	Average. Mol. Wt. (kDa)^a^	Conjugate Composition Antigen/carrier^b^	Antigen/carrier Mol. Wt. (kDa)
PfCSP-EPA	MV-8058	TE	259–4,238	1,075	1.72	32,578/66,983
PfCSP-EPA	MV-8073	Am	223–3,390	1,140	2.1	32,578/66,983
PfCSP-EPA	MV-8076	GA	291–1,840	797	1.6	32,578/66,983
PfCSP-EPA	MV-8063	ADH	220–1,590	654	1.45	32,578/66,983
Pfs25-EPA	MV- 8079	TE	184–1914	743	4.13	18,735/66,983
Pfs25-EPA	MV-8080	Am	209–1878	1510	2.37	18,735/66,983
Pfs230D1-EPA	MV- 8090	TE	170–1817	648	2.53	21,854/66,983
Pfs230D1-EPA	MV-8093	Am	188–1767	690	2.08	21,854/66,983
Pfs25-TT	MV-8081	TE	510–6,844	2,473	9.15	18,735/150,551
Pfs25-CRM197	MV-8085	TE	283–2,204	904	3.76	18,735/58,413

^a^: data from SEC-MALS analysis.

^b^: data obtained by Amino Acid Analysis of the conjugates and monomers.

TE: thioether chemistry; Am: Amide chemistry; GA: Glutaraldehyde chemistry; ADH: Adipic acid dihydrazide chemistry.

**Fig 1:**
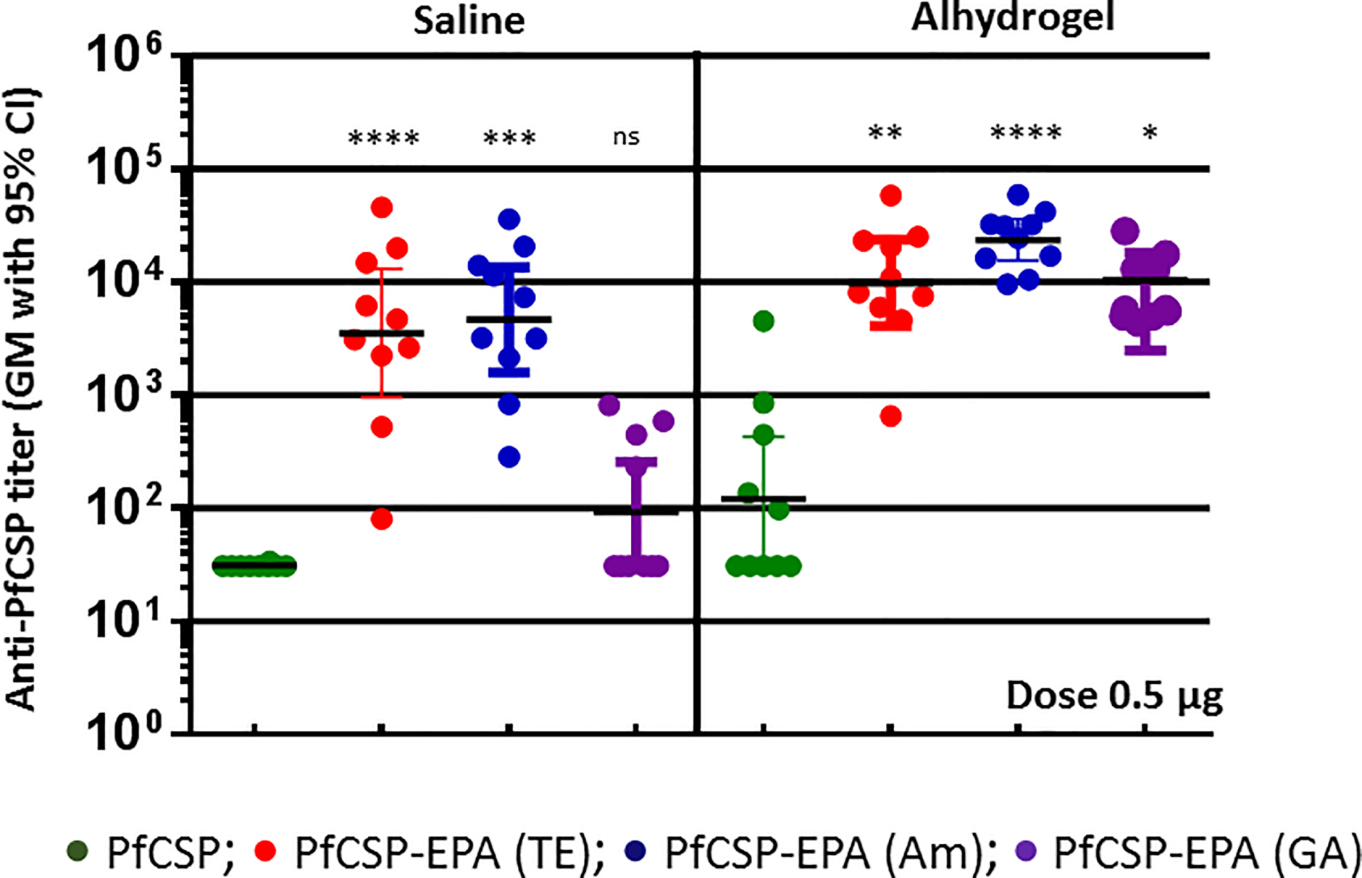
Anti-CSP antibody titer of EPA conjugates of PfCSP synthesized by thioether (TE), Amide (Am) and Glutaraldehyde (GA) conjugation chemistry. Mice were immunized with conjugates formulated in saline or Alhydrogel. Sera were analyzed on Day 42 after vaccination on Day 0 and 28. Dose: 0.5 μg/mouse.

Immunogenicity of the conjugates formulated in saline or Alhydrogel was evaluated in mice, using a schedule of two vaccinations (on Days 0 and 28) followed by collection of sera on Day 42. Sera from mice immunized with PfCSP monomer in saline showed very low titer that was enhanced by formulation in Alhydrogel (Fig 1). Conjugates synthesized by TE and amide chemistries showed significant enhancement in immunogenicity in saline compared to unconjugated PfCSP, demonstrating an effect of conjugation on immunogenicity. Conjugates made using TE and amide chemistries showed similarly strong mean titers (~100 –fold higher compared to unconjugated antigen) while conjugates prepared with GA chemistry showed the least enhancement (~5 fold). Formulation in Alhydrogel resulted in further enhancement of the titer for all conjugates, with significantly higher titers to conjugated versus unconjugated antigen, but no significant difference between the three conjugates.

**Fig 2:**
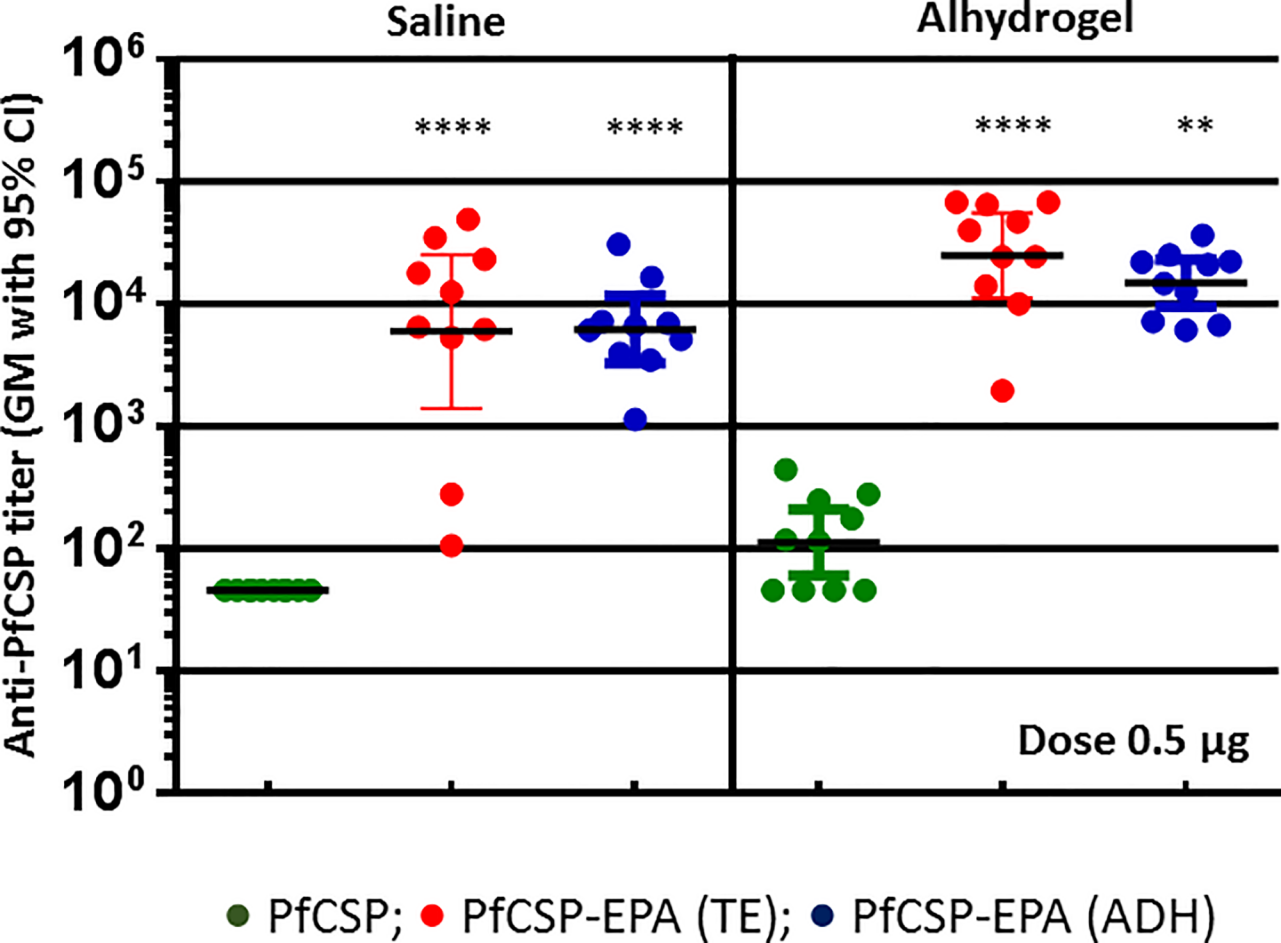
Anti-CSP antibody titer of EPA conjugates of PfCSP synthesized by thioether (TE) and Adipic acid Dihydrazide (ADH) conjugation chemistry. Mice were immunized with conjugates formulated in saline or Alhydrogel. Sera were analyzed on Day 42 after vaccination on Day 0 and 28. Dose: 0.5 μg/mouse.

A second mouse study compared PfCSP conjugates prepared using ADH and TE chemistries. Again, formulation in Alhydrogel enhanced the immunogenicity of the unconjugated antigen marginally, whereas both TE and ADH conjugates showed a significant increase in immunogenicity compared to unconjugated antigen, both in saline and Alhydrogel (Fig 2). Immunogenicity of the two conjugates was similar, both when formulated in saline and in Alhydrogel. In summary, all PfCSP conjugates enhanced immunogenicity versus monomer antigen; although some immunogenicity differences were observed between conjugates in saline, conjugates formulated in Alhydrogel yielded similar titers (Figs 1 and 2). The average molecular weight of PfCSP-EPA conjugates synthesized by different chemistries ranged between 654 and 1140 kDa (with a mean 917 ± 230 kDa), and similar molecular weight distribution was observed by size exclusion chromatography (S2 Fig).

**Fig 3:**
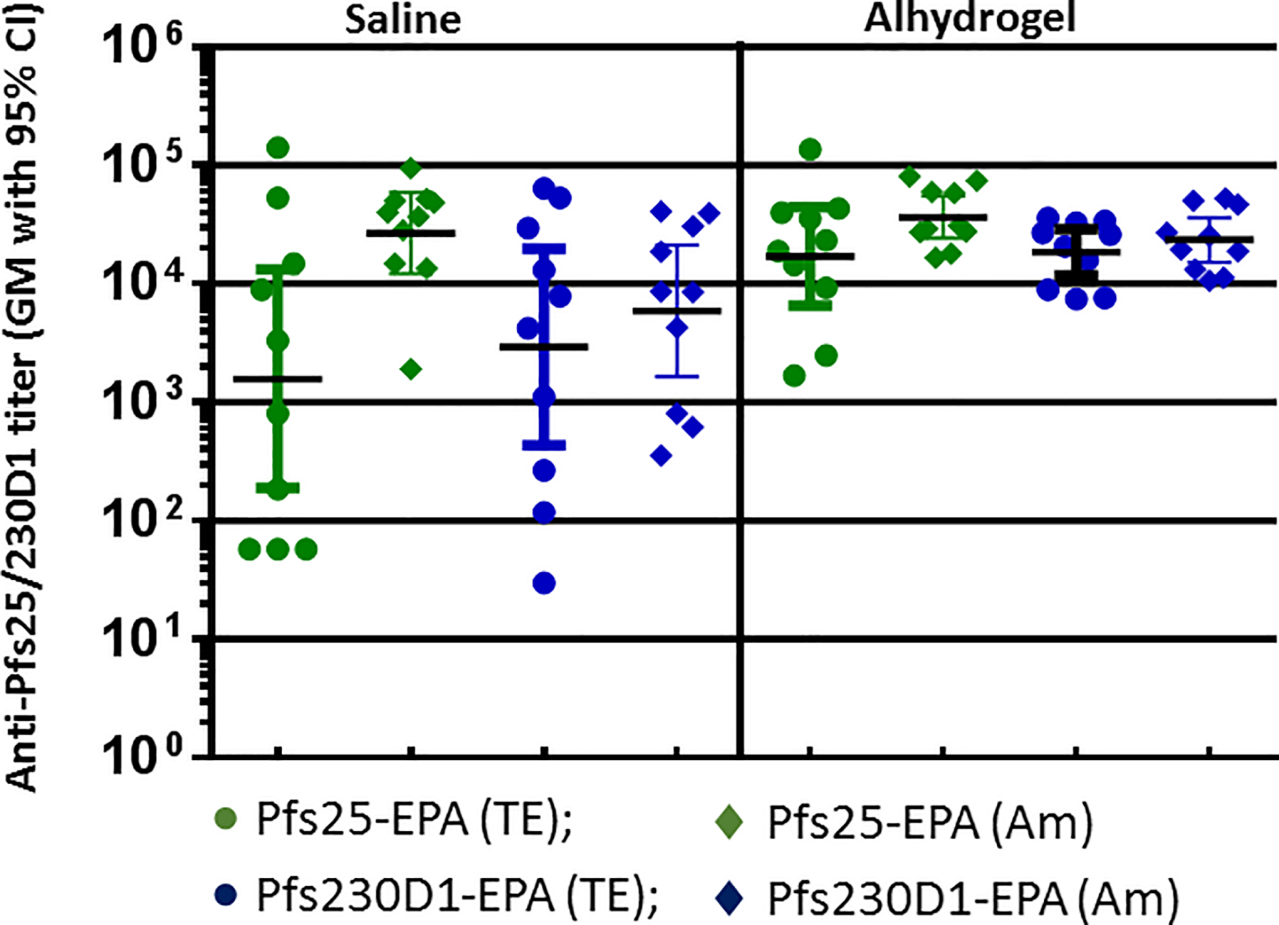
Anti-Pfs25 or Pfs230D1 antibody titer of EPA conjugates of Pfs25 and Pfs230D1 synthesized by thioether (TE) and Amide (Am) conjugation chemistry. Mice were immunized with conjugates formulated in saline or Alhydrogel. Sera were analyzed on Day 42 after vaccination on Day 0 and 28. Dose: 0.5 μg/mouse.

We then examined EPA conjugates of two TBV antigens, Pfs25 and Pfs230D1, synthesized by TE and Amide chemistries in mouse immunogenicity studies. As observed with the PfCSP conjugates, differences in titers observed in saline formulations disappeared when the conjugates were formulated in Alhydrogel (Fig 3). We observed some increases (though not significant) in titer on formulating in Alhydrogel, but the main effect of Alhydrogel was a reduced variance of titers within groups. Neither Pfs25 nor Pfs230D1 conjugates showed any significant difference based on conjugation chemistry when formulated in saline or Alhydrogel. Since the immunogenicity of the conjugates tested did not differ significantly based on the specific chemistry used for crosslinking, especially in Alhydrogel, we decided to use TE chemistry as the preferred method of conjugation. This approach has the benefit of two-step process: in the first step, antigen and carrier are separately modified, purified and characterized; in the second step, the two are combined in appropriate ratios to enable crosslinking. This allows for better control of the process, characterization of the intermediates, and scale up [45].

### Effect of adjuvants and carriers

**Fig 4:**
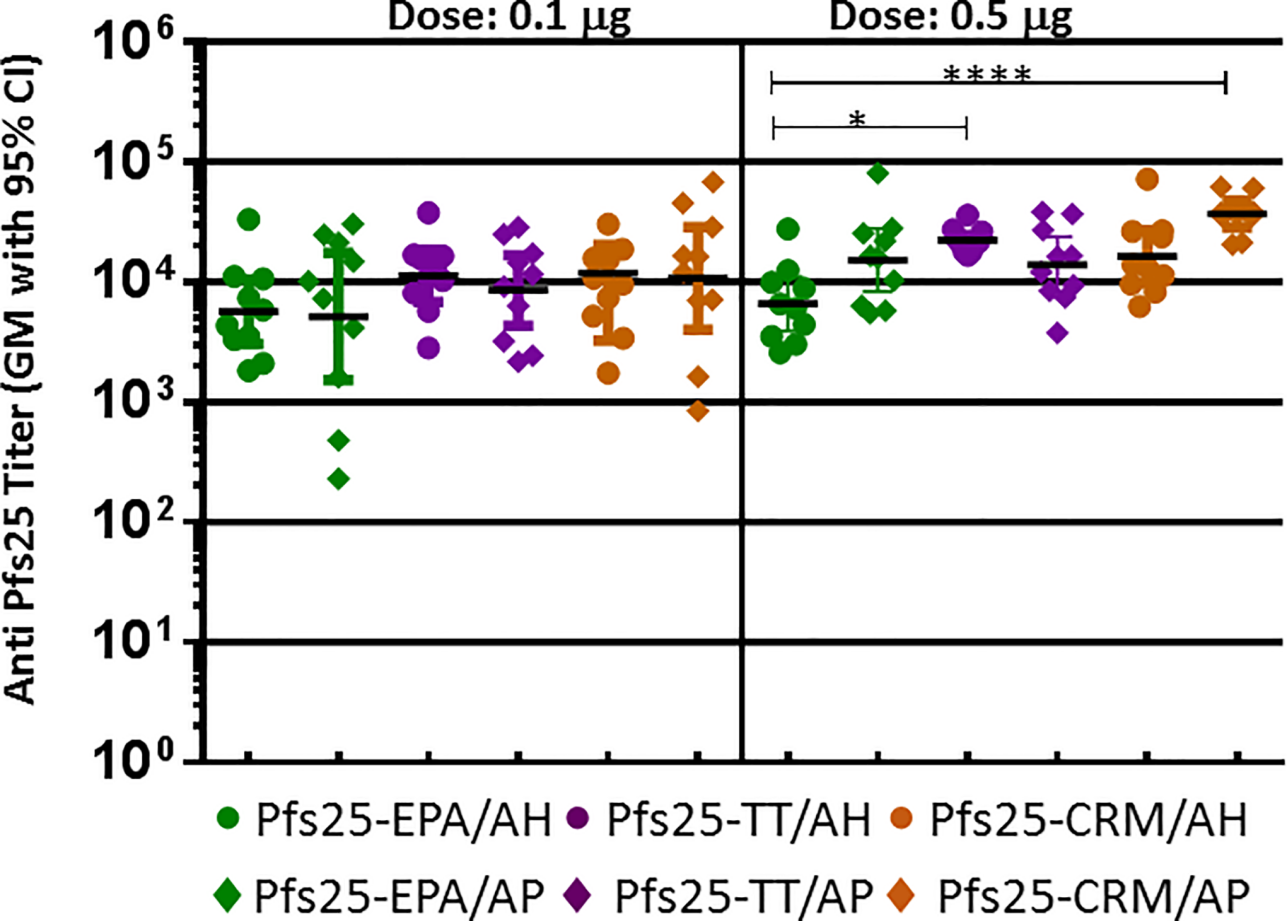
Anti-Pfs25 antibody titer of EPA, TT or CRM conjugates of Pfs25 synthesized by thioether (TE) chemistry formulated in either Alhydrogel (AH) or AdjuPhos (AP). Immune sera were collected from Day 42 after two vaccinations were analyzed. Dose: 0.5 μg/mouse.

Adjuvants play a key role in initiating the immune response against subunit vaccines and have been an essential component of vaccines, especially those comprised of purified recombinant protein antigens. Alum, the most common adjuvant in approved adjuvanted vaccines [56] and the only adjuvant approved in the US until Cervarix (an HPV vaccine containing AS04 approved in 2009 [57]), has been shown to be effective and safe while promoting Th2-mediated humoral immune responses. In addition to adjuvants, carrier proteins used for conjugation can influence the immunogenicity of the antigen. TT and CRM197 are two protein carriers used in a number of approved polysaccharide conjugate vaccines [21]. We conjugated malaria antigens to these carrier proteins and employed both thioether and amide chemistry to synthesize the conjugates of TT; the conjugates did not show any difference in their immunogenicity based on the conjugation chemistry (S3 Fig). Since we did not see any significant chemistry-dependent differences in immunogenicity for the TT or EPA conjugates, CRM197 conjugates were prepared using TE chemistry only. We evaluated EPA, TT and CRM conjugates of Pfs25 in combination with the commercially available adjuvants, Alhydrogel and AdjuPhos. These two alum-based adjuvants approved for use in human vaccines are available in wet gel suspensions, and differ in their ability to adsorb antigens due to their net charge at neutral pH: Alhydrogel has net positive charge while AdjuPhos has a net negative charge. All formulations were found to fully bind to Alhydrogel and AdjuPhos before testing them in mouse immunogenicity studies. Each conjugate, either in Alhydrogel or AdjuPhos, elicited very similar antibody titers (Fig 4), assessed at two different doses of the antigen (0.1 μg and 0.5 μg). No significant difference in titer was observed between Alhydrogel and AdjuPhos formulations for any of the three conjugates. Though EPA, TT and CRM conjugates elicited similar Pfs25 titers at the lower dose, the immunogenicity of EPA conjugate in Alhydrogel was significantly lower than that of the TT and CRM conjugates in Alhydrogel and AdjuPhos respectively, indicating that the latter two carriers may have beneficial effects.

### Particle size and structure of conjugates

Most successful vaccines are inherently particulate in nature or are made into particulate structures by association with particulate matter or adjuvants [58]. Though the effect of particulate structure on the enhanced immunogenicity of antigens is not fully understood, it is thought to facilitate internalization of the associated antigen by antigen-presenting cells, transport of the delivered antigen to the secondary lymph nodes either by passive flow or mediated by DCs, and depot effect preventing diffusion of soluble antigen [58–62]. We examined whether our conjugates form particulate structures and if so, whether there is any effect of size on immunogenicity.

The random crosslinking method employed to generate the conjugate allows us to produce conjugates of large molecular weight. However, this procedure also results in conjugates with a wide molecular weight distribution. Therefore, immunogenicity observed for these conjugates will be an average of small to large conjugates, and thus the effect of size on immunogenicity may not be apparent. To evaluate the effect of particle size of the conjugates on their immunogenicity, we prepared conjugates with sufficiently large differences in average size and tested their immunogenicity. Our conjugation method allows preparation of conjugates with different average molecular weight by controlling the concentration of the conjugation reaction.

**Fig 5:**
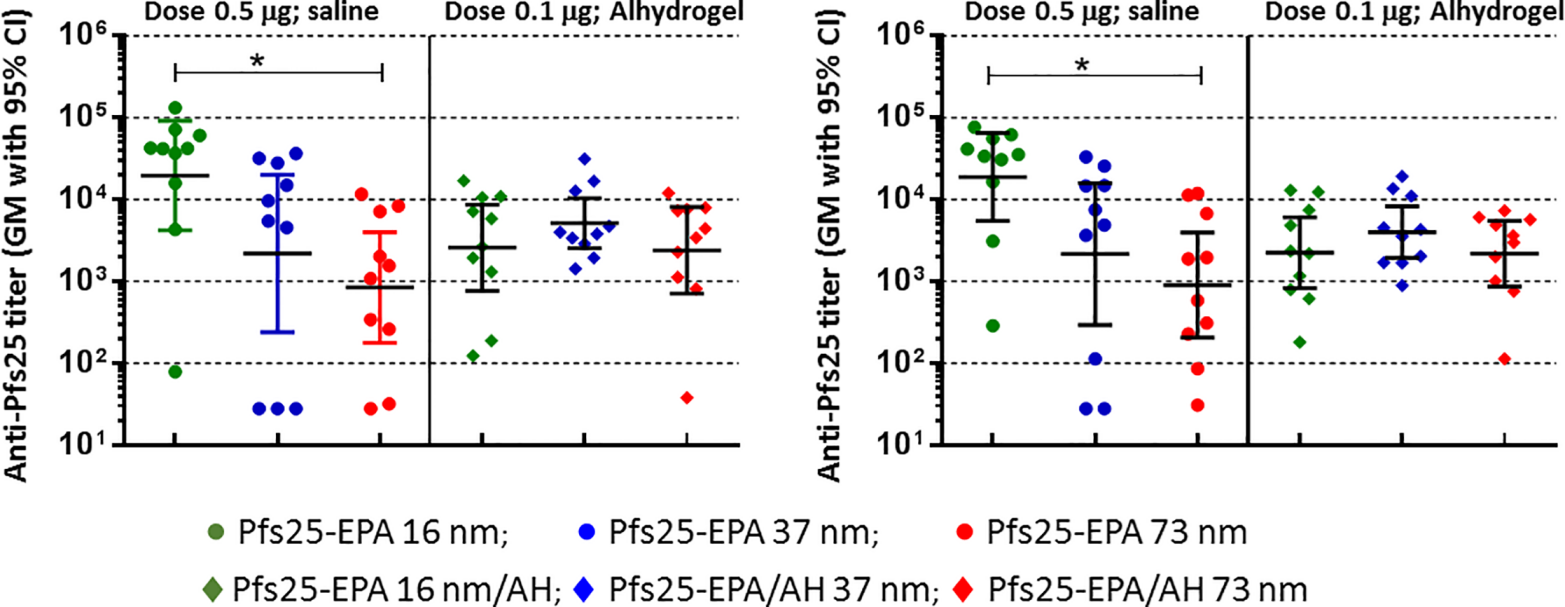
Anti-Pfs25 antibody titer of EPA conjugates of Pfs25 with varying average molecular weight and particle size, synthesized by thioether (TE) chemistry, formulated in either saline or Alhydrogel (AH). Immune sera collected on Day 42 (left panel) or 70 (right panel) were analyzed by ELISA. Vaccinations on Days 0 and 28. Doses: 0.5 μg or 0.1 μg/mouse/injection as indicated.

We synthesized Pfs25-EPA conjugates with varying average molecular weight, and selected SEC fractions that were sufficiently different in average size for further analysis of their immunogenicity (Table 2, S4 Fig). We selected 3 different conjugates that ranged in size and average molecular weight between 16 nm -73 nm and 743 kDa– 7,590 kDa respectively, and had an average diameter of 16, 37 and 73 nm respectively measured by Dynamic Light Scattering. We examined the immunogenicity of these conjugates in two different conditions, formulated in saline or in Alhydrogel, to assess any differences based on the size of the conjugate. We used the 0.5 μg dose for the saline formulated conjugates whereas a lower dose (0.1 μg) was used for Alhydrogel formulated conjugates, in order to distinguish any small difference in immunogenicity between these conjugates. Alhydrogel generally yields a strong immune response in mice which may mask any minor difference between the conjugates when given at higher doses. In saline, conjugates (0.5 μg doses) with an average size of 16 nm (molecular weight 743 kDa) showed the highest level of antibody titer, and titer decreased as particle size increased, with significant differences between 16 nm and 73 nm size (molecular weight 7,590 kDa) particles (Fig 5). In Alhydrogel (0.1 μg doses), all three conjugates had similar antibody titers. A similar trend was observed when the sera collected on Day 70 were analyzed (Fig 5, right panel). Presumably, conjugates, regardless of size, adsorb to relatively large Alhydrogel particles (~0.5–20 μm) [61], effectively nullifying the size-related differences of the unformulated conjugates.

**Table 2:** Physico-chemical parameters of Pfs25 -EPA conjugates with different molecular weight and particle size used in the mice study evaluating the effect of size on the immunogenicity (Fig 5).

Conjugate	Lot #	Conjugation Chemistry	Mol. Wt. distribution (kDa)^a^	Average. Mol. Wt. (kDa)^a^	Conjugate Composition: Antigen/total protein^b^	Particle Diameter ^c^
Pfs25-EPA	MV- 8079	TE	184–1,914	743	0.53	16 ± 2
Pfs25-EPA	MV- 8100	TE	225–3,404	1250	0.45	37 ± 2
Pfs25-EPA	MV- 8099	TE	1935–23,881	7590	0.43	73 ± 3

^a^: data from SEC-MALS analysis.

^b^: data obtained by Amino Acid Analysis of the conjugates and monomers.

^c^: data from dynamic light scattering.

**Fig 6:**
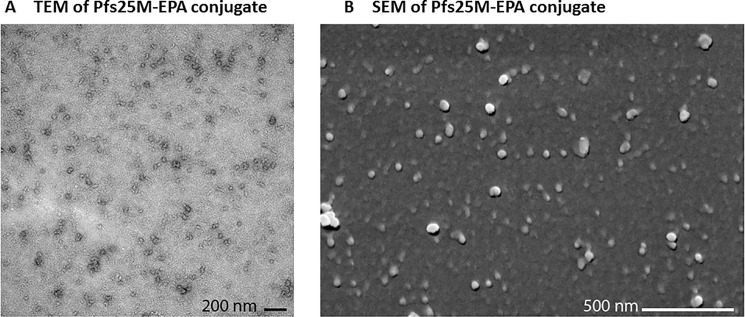
Transmission electron microscopy (A) and scanning electron microscopy images of Pfs25-EPA conjugate. Scale bars, A: 200 nm, B: 1.0 um.

We further examined if protein conjugates form nanoparticle structures by electron microscopy. TEM (Fig 6A) and SEM (Fig 6B) images of one of the Pfs25-EPA conjugates clearly show particle structures with nanoparticle dimensions and largely spherical morphology.

## Conclusions

We demonstrate here that protein-protein conjugation is a robust strategy to enhance the immunogenicity of poorly immunogenic antigens. In this study, we examined three different poorly immunogenic malaria antigens, conjugated to three different protein carriers, to evaluate the effect of conjugation on immunogenicity. In all cases, we have seen significant increases in immunogenicity upon conjugation to a carrier protein. We tested four different conjugation chemistries, all of which appear to be suitable; no significant differences in immunogenicity were observed based on conjugation chemistry used, except for the glutaraldehyde conjugates, which showed lower immunogenicity compared to other conjugates in saline.

These results show that protein-protein conjugation is a broadly applicable strategy offering a robust platform for vaccine development. Conjugates can be synthesized by well-established synthetic chemistry approaches and can tolerate significant levels of variations in the conjugate molecular weight and distribution without significantly affecting their immunogenicity (S5A–S5D Fig). As the data presented indicate, a variety of options are available to generate conjugates of antigen to carriers. There are numerous other chemical approaches that were not tested here but may be viable as well. In addition to EPA, TT and CRM197 carriers tested in this study, a variety of potential carriers are available including proteins, nanoparticles, and virus-like particles, among others. From the data on various conjugates, it is apparent that conjugation can augment the immunogenicity of antigens. This may be attributable to T cell epitopes from the carrier proteins and the particulate nature of the conjugate which may be more efficiently internalized and processed by antigen-presenting cells. Size of the particle can be an important parameter for immunogenicity. Limited data presented here suggest that smaller particles may be more immunogenic for the EPA conjugates of Pfs25, though a significant decrease in immunogenicity could be observed only when the average particle size was increased considerably. However, the size dependence of a particulate vaccine is complex and there is contradictory evidence about its effect on immunogenicity [61]. This may be due to the fact that antigen delivery and immune responses employ redundant mechanisms that involve passive transport to lymph nodes to active transport by antigen-presenting cells that can handle particles of varying sizes and morphologies at varying efficiencies, nonetheless resulting in the generation of an immune response. Even with the data presented here, it is apparent that particle size alone does not explain the immunogenicity or lack of it. For example, PfCSP-EPA (GA) in saline (Fig 1) showed low immunogenicity, even though the same conjugate made by TE chemistry with higher molecular weight (Table 1) showed higher immunogenicity. Similarly, Pfs25-TT showed higher immunogenicity compared to Pfs25-EPA made by TE chemistry, though TT conjugate had a higher molecular weight (Fig 4, Table 1). This shows that particle size dependence of immunogenicity cannot be easily generalized across conjugates made by different chemistries and carriers. Additional effects such as chemistry dependent antigen modifications, quantity and quality of T-cell epitopes provided by carriers, and possibly other factors can influence the immune response against the conjugated antigen (48). Each system needs to be evaluated separately as done in this study for the Pfs25-EPA conjugates, to optimize each conjugate for maximal immunogenicity. More extensive studies, using particles with narrow size distribution and assessing their mode of transport to the lymph nodes, may give more insight into the mechanism of size dependency.

## Supporting information

S1 Fig SDS-PAGE and Western Blot analysis of PfCSP conjugates synthesized by four different chemistries. (A) SDS-PAGE of conjugates synthesized with different chemistries (lanes 2–5) compared to PfCSP (lane 6) and EPA (lane 7), along with molecular weight markers in lanes 1 & 8. (B) Western blot of the conjugates developed by staining with a monoclonal antibody, 1G2, against PfCSP. Conjugates in Lanes 2–5 are compared with PfCSP in lane 6 and EPA in lane 7.
(TIF)

S2 Fig Size exclusion chromatography of PfCSP-EPA conjugates synthesized by four different chemistries. Conjugates were analyzed using G5000PWxl column and PBS as running buffer with monitoring A_280_. Average molecular weight and molecular weight distribution (Table 1) were analyzed by multi-angle light scattering (SEC-MALS). * corresponds to residual EDTA from conjugation buffer.
(TIF)

S3 Fig Anti-Pfs25 antibody titer of TT conjugates of Pfs25 synthesized by thioether (TE) and amide (Am) chemistry formulated in Alhydrogel®. CD-1 mice (groups of 10) were immunized with 0.5 μg dose (in terms of Pfs25) of conjugates formulated in Alhydrogel® by intramuscular injection on days 0 and 28. Sera were collected on day 42 and assayed for anti-Pfs25 antibody titer.
(TIF)

S4 Fig Size exclusion chromatography of Pfs25-EPA conjugates used for particle size dependence of immunogenicity. Conjugates were analyzed using G5000PWxl column and PBS as running buffer with monitoring A_280_. Average molecular weight and molecular weight distribution were analyzed by multi-angle light scattering (SEC-MALS).
(TIF)

S5 Fig **Size exclusion chromatography of three different batches of Pfs25-EPA conjugates (A) and their anti-Pfs25 antibody titer, (B) evaluated in 6 independent animal studies demonstrating the reproducibility of immunogenicity. (C and D) Demonstration of the reproducibility of synthesis.** SEC of the conjugates were analyzed on a G5000PWxl size exclusion column using PBS as running buffer and monitored by A_280_. Average molecular weight and molecular weight distribution were analyzed by multi-angle light scattering (SEC-MALS). For in vivo experiments, CD-1 mice (groups of 10) were immunized with 0.5 μg dose (in terms of Pfs25) of conjugates formulated in Alhydrogel by intramuscular injection on days 0 and 28. Sera were collected on day 42 and assayed for anti-Pfs25 antibody titer. Immunogenicity data were generated from 6 independent in vivo studies. Statistical analysis (Kruskal-Wallis analysis followed by Dunn multiple comparator test) of antibody titer showed no significant difference in titer from the six different experiments. Size exclusion chromatography of two different batches of Pfs25-CRM197 (C) and two different batches of Pfs25-TT (D) conjugates, demonstrating the reproducibility of conjugate synthesis. Conjugates of Pfs25 with CRM197 and TT were synthesized by thioether chemistry and were analyzed by SEC using G5000PWxl column and PBS as running buffer with monitoring A_280_.
(TIF)
