# Supplementary figures and images for: Protein-protein conjugate nanoparticles for malaria antigen delivery and enhanced immunogenicity
Source: PLoS One. 2017 Dec 27;12(12):e0190312. doi: 10.1371/journal.pone.0190312 (PMC5744994; doi:10.1371/journal.pone.0190312)

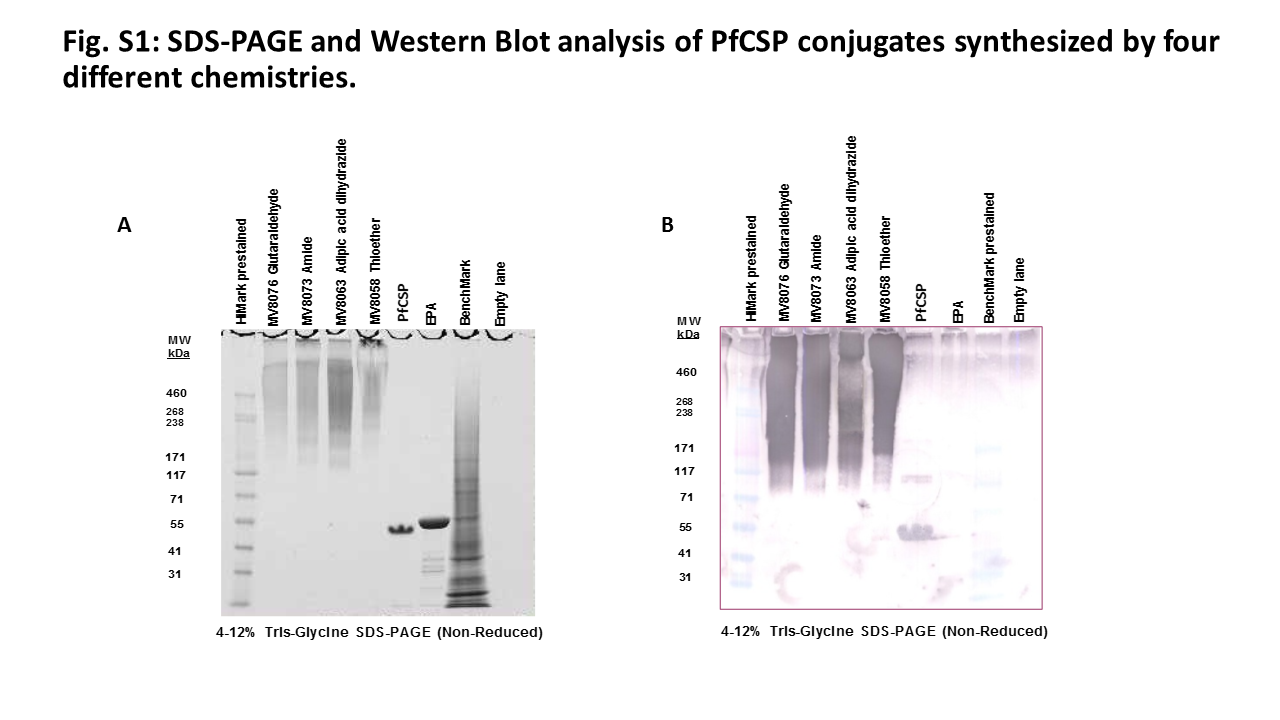

Supplement: S1 Fig — (A) SDS-PAGE of conjugates synthesized with different chemistries (lanes 2–5) compared to PfCSP (lane 6) and EPA (lane 7), along with molecular weight markers in lanes 1 & 8. (B) Western blot of the conjugates developed by staining with a monoclonal antibody, 1G2, against PfCSP. Conjugates in Lanes 2–5 are compared with PfCSP in lane 6 and EPA in lane 7. (TIF) [file pone.0190312.s001.TIF]

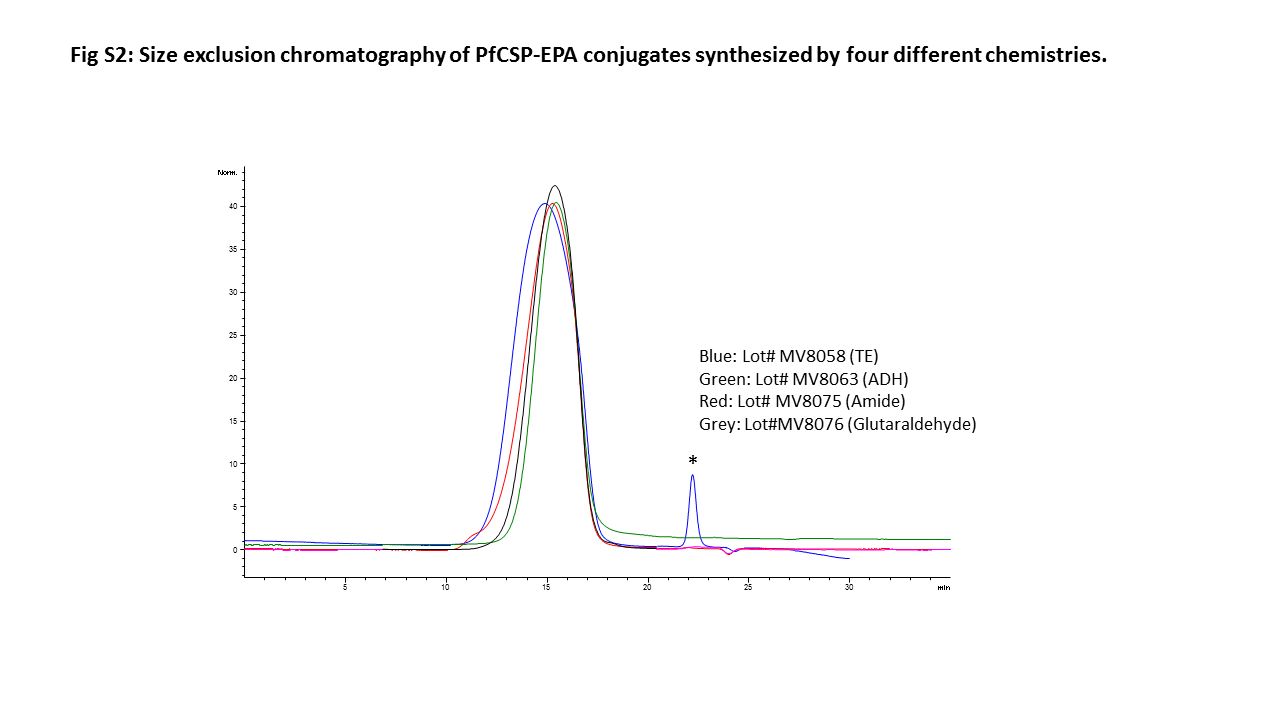

Supplement: S2 Fig — Conjugates were analyzed using G5000PWxl column and PBS as running buffer with monitoring A280. Average molecular weight and molecular weight distribution (Table 1) were analyzed by multi-angle light scattering (SEC-MALS). * corresponds to residual EDTA from conjugation buffer. (TIF) [file pone.0190312.s002.TIF]

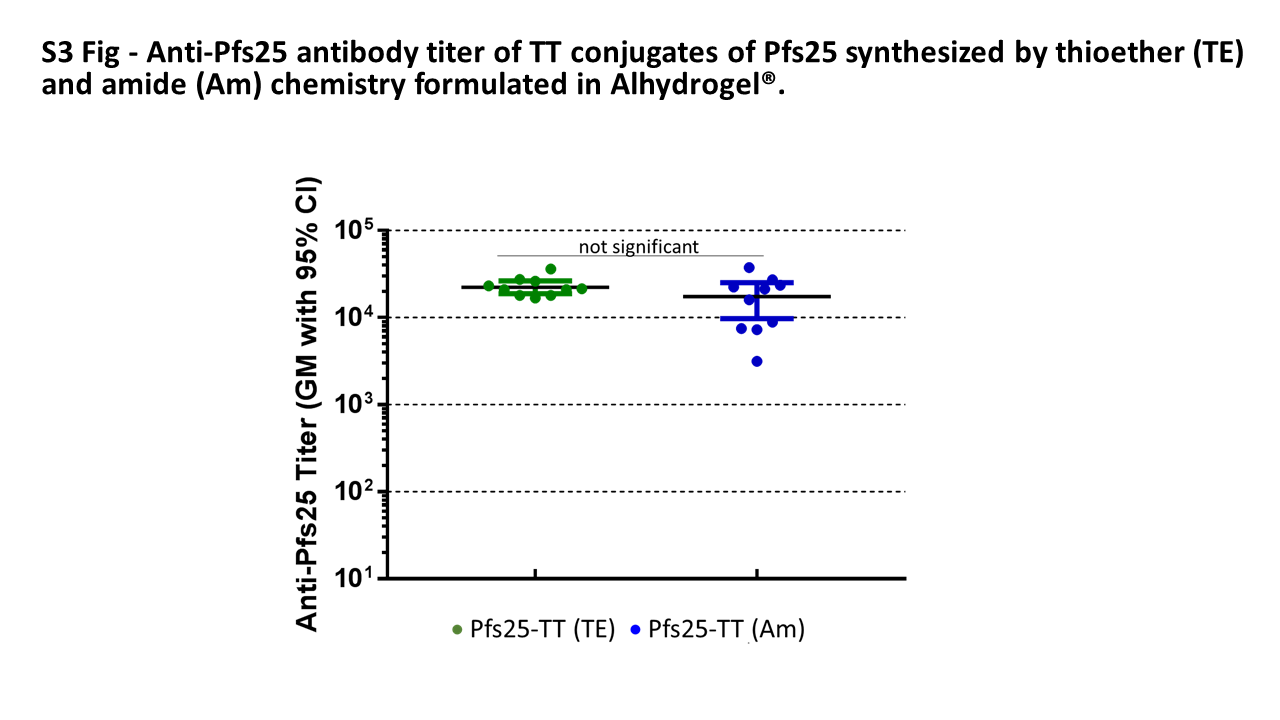

Supplement: S3 Fig — CD-1 mice (groups of 10) were immunized with 0.5 μg dose (in terms of Pfs25) of conjugates formulated in Alhydrogel® by intramuscular injection on days 0 and 28. Sera were collected on day 42 and assayed for anti-Pfs25 antibody titer. (TIF) [file pone.0190312.s003.TIF]

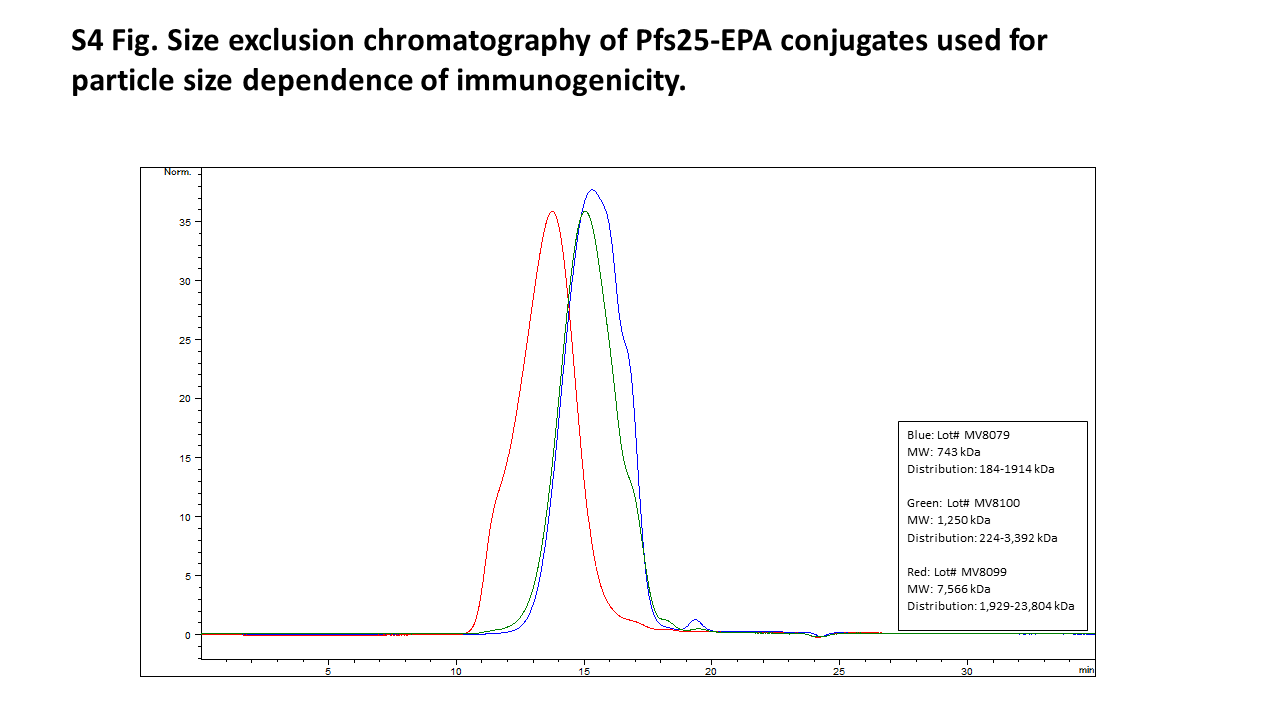

Supplement: S4 Fig — Conjugates were analyzed using G5000PWxl column and PBS as running buffer with monitoring A280. Average molecular weight and molecular weight distribution were analyzed by multi-angle light scattering (SEC-MALS). (TIF) [file pone.0190312.s004.TIF]

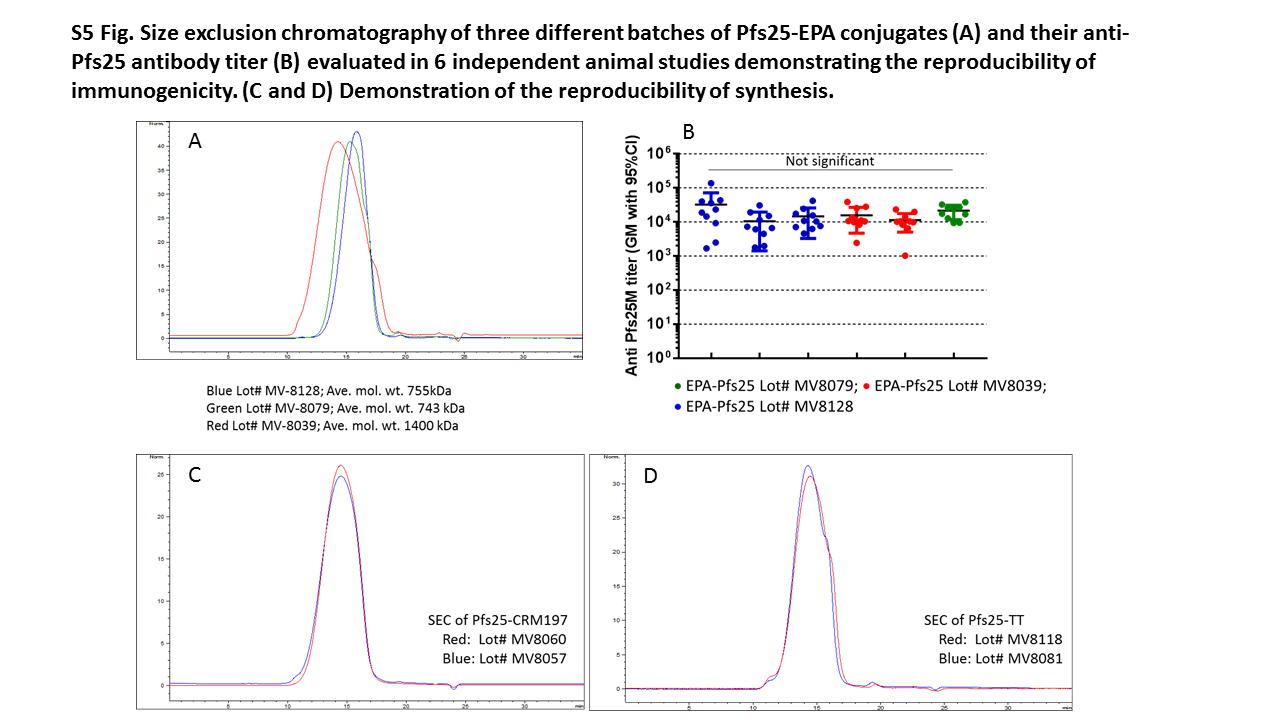

Supplement: S5 Fig — Size exclusion chromatography of three different batches of Pfs25-EPA conjugates (A) and their anti-Pfs25 antibody titer, (B) evaluated in 6 independent animal studies demonstrating the reproducibility of immunogenicity. (C and D) Demonstration of the reproducibility of synthesis. SEC of the conjugates were analyzed on a G5000PWxl size exclusion column using PBS as running buffer and monitored by A280. Average molecular weight and molecular weight distribution were analyzed by multi-angle light scattering (SEC-MALS). For in vivo experiments, CD-1 mice (groups of 10) were immunized with 0.5 μg dose (in terms of Pfs25) of conjugates formulated in Alhydrogel by intramuscular injection on days 0 and 28. Sera were collected on day 42 and assayed for anti-Pfs25 antibody titer. Immunogenicity data were generated from 6 independent in vivo studies. Statistical analysis (Kruskal-Wallis analysis followed by Dunn multiple comparator test) of antibody titer showed no significant difference in titer from the six different experiments. Size exclusion chromatography of two different batches of Pfs25-CRM197 (C) and two different batches of Pfs25-TT (D) conjugates, demonstrating the reproducibility of conjugate synthesis. Conjugates of Pfs25 with CRM197 and TT were synthesized by thioether chemistry and were analyzed by SEC using G5000PWxl column and PBS as running buffer with monitoring A280. (TIF) [file pone.0190312.s005.TIF]
